# Association between Intensity Levels of Physical Activity and Glucose Variability among Children and Adolescents with Type 1 Diabetes

**DOI:** 10.3390/ijerph20021623

**Published:** 2023-01-16

**Authors:** Jason R. Jaggers, Kristi M. King, Timothy McKay, Ryan J. Dyess, Bradly J. Thrasher, Kupper A. Wintergerst

**Affiliations:** 1 Wendy Novak Diabetes Center, Division of Pediatric Endocrinology, School of Medicine, University of Louisville, Louisville, KY 40202, USA; 2 Department of Health and Sport Sciences, University of Louisville, Louisville, KY 40292, USA; 3 Department of Kinesiology and Health Sciences, Georgetown College, Georgetown, KY 40324, USA; 4 Norton Children’s Medical Group, University of Louisville School of Medicine, Louisville, KY 40202, USA

**Keywords:** clinical exercise, glycaemia, accelerometer, sport medicine, glycemic variability

## Abstract

Studies would indicate a reduction in hemoglobin A1c levels following moderate and/or vigorous physical activity (PA) for people managing diabetes. However, prior investigations rarely looked at glucose variability in an adolescent population. Purpose: The purpose of this investigation was to test the relationship between physical activity intensity levels and glucose variability in a sample of adolescents with type 1 diabetes mellitus, and if the amount of time accumulated for each intensity level is predictive of changes in glucose variability. Methods: Glucose variability was determined using continuous glucose monitor data and physical activity intensity time was retrieved through Fitabase^®^. Both glucose and physical activity data were collected over a two-week timeframe. Data analysis was completed using Pearson’s correlation and a simple linear regression with a *p*-value of 0.05 to determine significance. Results: A significant inverse relationship was observed (*p* = 0.04) between glucose variability and average minutes of daily moderate-intensity activity (r = −0.59), as well as moderate and vigorous physical activity (MVPA) combined (r = −0.86; *p* = 0.03). A simple linear regression indicated that only MVPA was a significant predictor of glucose variability (β = −0.12; 95% CI: −0.23–−0.01, *p* = 0.03). Conclusion: These data demonstrated that the total amount of daily physical activity is important when properly managing type 1 diabetes mellitus, but time spent in MVPA over two weeks may have an inverse relationship with glucose variability in children and adolescents over a span of two weeks.

## 1. Introduction

It has been well established in multiple populations with and without diabetes that daily bouts of physical activity and/or routine exercise promote positive changes in circulating glucose levels [1,2,3,4,5]. For people managing type 1 diabetes mellitus, these changes are often dependent on the duration and intensity of activity [4,6]. Current research has examined the glucose response for people managing type 1 diabetes mellitus following exercise interventions set at specific intensities and durations [7,8]. These studies have helped lead to the development of recommended precautionary measures one should take prior to being physically active [4,8]. However, the majority of these investigations took place in a controlled environment with older adult populations. Little is known regarding the impact real-world stop-and-go activities of varying intensities, such as sporting competitions or practices, has on the glucose response in young adults or children managing type 1 diabetes mellitus. To our knowledge, only one research study has looked specifically at daily intensity levels of exercise or physical activity and its impact on glucose values hours later, including overnight which could lead to the risk of nocturnal hypoglycemia [9]. Even less is clear regarding glucose fluctuations when multiple bouts of daily activity are a part of one’s lifestyle. 

The American Diabetes Association’s position statement “Type 1 Diabetes through the life span” has reported clear and concise recommendations for health that include routine physical activity and exercise [10]. This statement provides a detailed overview of the research that has been published with a consensus on exercise management for individuals with type 1 diabetes mellitus who exercise regularly, including glucose targets for safe and effective exercise, and nutritional and insulin dose adjustments to protect against exercise-related glucose fluctuations [10]. Other position stands published by the American Diabetes Association that are specific to physical activity and diabetes management have provided similar guidance but unfortunately, these recommendations are relatively general and based primarily on research studies with adults managing type 2 diabetes mellitus [2,4]. It is imperative that individuals managing type 1 diabetes mellitus have recommendations that are specifically tailored to their individual needs. Providers have been increasingly turning to diabetes technology to better serve their patients, and the use of activity monitoring technology would be another solid approach. Incorporating new wearable devices such as an activity monitor into standard medical care would be an innovative approach to improving diabetes management for adolescents with type 1 diabetes mellitus who engage large amounts of physical activity or exercise regularly throughout the week. As stated previously, more research is still needed to help aid in the recommendations for individuals managing type 1 diabetes mellitus when participating in bouts of activities that are often of varying intensity levels. This has led to questions regarding glucose variability following multiple days of routine exercise or physical activity habits. 

Glucose variability refers to blood glucose oscillations throughout the day and is a common metric used in diabetes management that has become a valuable tool to help physicians determine glycemic control and risk of complications when making decisions regarding their patients care [11]. Glucose variability has been proposed as a risk factor for microvascular complications [12] and has been found to be useful to predict risk of hypoglycemia in patients with type 1 diabetes mellitus [13]. With the help of continuous glucose monitors, glucose variability is one of many tools to investigate the impact of changes in a patient’s diabetes care such as insulin adjustments and carbohydrate ratios. The majority of research completed on the topic of glucose variability has focused on specific insulin regimens, carbohydrate intake, or other dietary variables [11]. 

Managing diabetes with insulin can often lead to extreme highs or lows when participating in recreational activities, exercise training, and/or routine sport participation even when following the necessary precautions that have been put into place with one’s healthcare provider and diabetes care team. Among the greatest obstacles to engaging in highly competitive sports or vigorous-intensity exercise/physical activities is maintaining safe glucose ranges to perform effectively at the highest level possible while also taking precautionary measures before and after to prevent hyper- or hypoglycemia. The results of a recent investigation using accelerometry simultaneously with continuous glucose monitor data for adolescent athletes with type 1 diabetes mellitus found that total minutes of moderate and vigorous-intensity physical activity accumulated throughout a single day is a predictor of hypoglycemic events overnight, and that vigorous-intensity physical activity alone is a predictor of the duration of each hypoglycemic event [9]. 

With the challenges faced by people engaging in daily physical activity and exercise while managing type 1 diabetes mellitus, the uncertainty of glucose control due to large swings in glucose variability can often act as a barrier to participating in routine exercise and/or recreational activities that are known to improve health and increase life longevity. There are also well-established psychological benefits that include stress management and improved mental state [14,15]. However, with limited research examining the impact of sporadic bouts of moderate and/or vigorous-intensity-level physical activity ranging from short to long time durations providing guidance on how to best manage the glucose response can be challenging for healthcare specialists and diabetes care teams. This is especially true for tips and recommendations to prevent nocturnal hypoglycemia since fluctuations in glucose levels can continue for hours following exercise or competitive events leading to dangerously low glucose levels overnight while asleep. 

Studies have consistently shown improvements in daily glucose and hemoglobin A1c (HbA1c) for both type 1 and type 2 diabetes mellitus following daily moderate and/or vigorous physical activity [4]. A limitation with prior investigations is that this relationship was only explored in adult populations and seldom looked at glucose variability. Whether similar results would be observed in a pediatric population with type 1 diabetes mellitus is unknown. To our knowledge, only one study has looked specifically at the relationship between glucose variability and intensity levels of exercise or physical activity duration [16]. However, prior investigations have not examined this relationship in a habitually active pediatric population with type 1 diabetes mellitus. The importance of insulin use to manage daily fluctuations in glucose makes it important to have a more clear understanding of the relationship between physical activity intensity and glucose to better anticipate how much and when insulin adjustments are needed prior to, during, and following intermittent bouts of activity and/or exercise at varying intensities. Therefore, the purpose of this investigation was to examine the relationship between physical activity intensity levels and glucose variability in a sample of adolescents with type 1 diabetes mellitus. 

## 2. Materials and Methods

### 2.1. Study Design

This study is a single-cohort design to assess the association between intensity of physical activity and glucose variability. The primary aim was to identify significant relationships between daily physical activity accumulation at varying intensity levels and glucose fluctuations over a two-week timeframe among children and adolescent athletes with type 1 diabetes mellitus. Participants who wore a continuous glucose monitor and an accelerometer consecutively for a period of two weeks or more were included in the data analysis.

### 2.2. Participants

Children and adolescents aged 10 to 17 years of age with type 1 diabetes mellitus receiving care at the Wendy Novak Diabetes Center at the Norton Children’s Hospital were invited to participate in this study. The inclusion criteria for this study were: established patients with type 1 diabetes mellitus cared for in the diabetes outpatient clinical office, daily use of a continuous glucose monitor, a current athlete in an organized sport at the middle or high-school level, and free of any musculoskeletal injuries within the last 12 months. Exclusion criteria included those who were non-English speaking and those who were under 18 without a parent or legal guardian to provide informed consent. This study was approved by the University’s Institutional Review Board and informed consent/assent was obtained prior to participation in this study (Approval # 18.0713).

### 2.3. Study Procedures

To assess physical activity frequency, intensity, and duration, participants were instructed to wear a consumer-based physical activity monitor on their wrist simultaneously with their continuous glucose monitor over a two-week time period while continuing their usual physical activity and sport participation behaviors. The Fitbit^®^ Charge 2 (Fitbit Inc., San Francisco, CA, USA) is a small, wireless device that fits within a wristband and uses a triaxial accelerometer to convert raw acceleration signals into counts. These counts are then applied to proprietary algorithms that provide estimates of steps/minute, physical activity level (sedentary, light, moderate, and vigorous), and energy expenditure. A Fitabase^®^ account was created for this study in order to obtain daily and minute-level Fitbit data that included estimated metabolic equivalents which quantified intensity levels. All participants were wearing a Dexcom^®^ brand continuous glucose monitor. Data from the continuous glucose monitor were extracted over the same two weeks in which the participant was wearing the activity monitor.

### 2.4. Data Analysis

Data from the activity monitors and continuous glucose monitor were exported from each device into an Excel spreadsheet that included participant demographics. Separate columns were added for continuous glucose monitor data including coefficient variation (CV) and standard deviation (SD) to determine glucose variability. Physical activity was analyzed using average time spent in light, moderate, and vigorous intensities. For this investigation, moderate and vigorous physical activity was analyzed combined and individually to test them independent of one another. To examine the relationships between intensity and glucose variability, Pearson’s correlation test was used. Intensity levels with significant relationships were further explored using a simple linear regression with glucose variability as the dependent variable. A *p*-value of <0.05 was considered statistically significant and final analysis was completed using statistical software SPSS version 25.

## 3. Results

**Figure 1:**
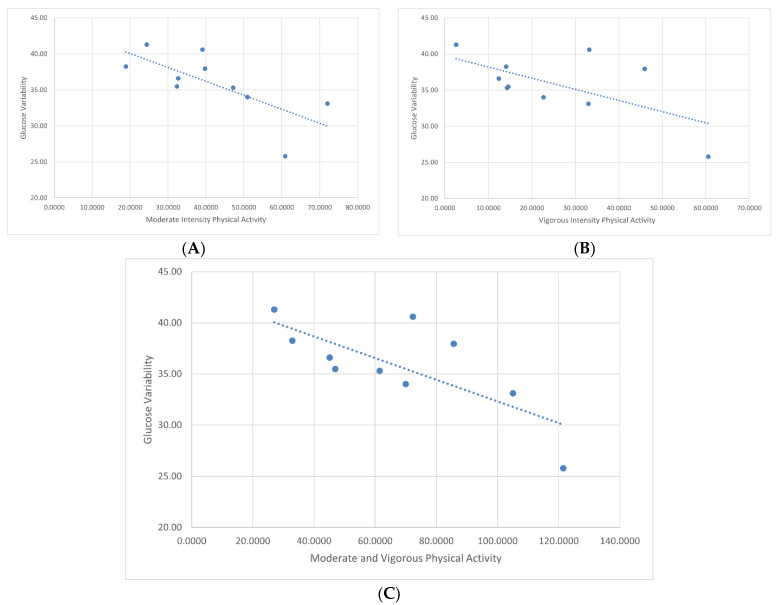
Association between glucose variability and intensity of physical activity. (**A**) Glucose variability versus moderate-intensity physical activity; Pearson’s correlation coefficient (r) = −0.59, *p* = 0.04; (**B**) glucose variability versus vigorous-intensity physical activity; r = −0.34, *p* = 0.42; (**C**) glucose variability versus combined moderate and vigorous physical activity; r = −0.86, *p* = 0.03.

Demographic information of study participants can be found in Table 1. Ten adolescents between the ages of 10 and 19 years old with a history of continuous glucose monitor use >6 months participated in this study, accumulating 140 days of data. Six managed their diabetes with an insulin pump, whereas four administered insulin with multiple daily injections. Participants accumulated an average of 23.46 ± 18.24 min of vigorous intensity, and 41.33 ± 15.29 of moderate-intensity activity per day. Average glucose variability over the two-week period was 36.64% ± 4.66 with an average of 64.43 ± 18.09 min of combined moderate and vigorous physical activity during that same timeframe. Pearson’s correlation showed that total minutes of daily moderate-intensity activity had a significant inverse relationship (r = −0.59; *p* = 0.04) with glucose variability, and correspondingly moderate and vigorous physical activity showed a stronger inverse relationship (r = −0.86; *p* = 0.03). Scatterplots of these relationships can be found in Figure 1.When placed in a simple linear regression (Table 2), only moderate and vigorous physical activity combined significantly predicted changes in glucose variability (β = −0.12, *p* = 0.03). Vigorous-intensity activity alone did not show a significant relationship with glucose variability.

## 4. Discussion

This investigation examined the associations between glucose variability and duration of moderate and vigorous-intensity physical activity in a sample of adolescents with type 1 diabetes mellitus. Results would suggest that duration of moderate and vigorous intensity levels during physical activity may be predictive of lower glucose variability over a two-week period. Compared to other investigations, these findings are in agreement with the reported findings from other studies looking at combined moderate and vigorous physical activity and diabetes management [4,8]. Even though we did not report on HbA1c changes due to the short nature of the data collection period, it could be inferred that by maintaining stable glucose through improved glycemic control one would expect to see an improvement in HbA1c. Findings from other investigators have reported the effects of moderate and vigorous physical activity on glucose levels in adolescents managing type 1 diabetes mellitus which have also indicated an increased risk of hypoglycemic events, especially overnight several hours following activity, was associated with the duration of moderate and vigorous physical activity [17,18]. It should be noted that these studies did not include measures of glucose variability over a two-week period. Specifically, there is a gap in the literature regarding the frequency, intensity, and duration of physical activity for adolescents with type 1 diabetes mellitus and its impact on glucose variability. It is still unknown how habitually active adolescents with type 1 diabetes mellitus respond in both the short- and long-term regarding glucose fluctuations.

Since all adolescents are encouraged to accumulate a total of 60 min of daily physical activity for known health benefits, it is of the utmost importance that those with type 1 diabetes mellitus be able to participate free from fear of diabetes-related complications. Current published reviews and consensus guidelines on management of type 1 diabetes mellitus for individuals who exercise regularly include specific glucose ranges for safe and effective exercise, as well as nutritional and insulin dose adjustments to protect against exercise-related hyper- and hypoglycemic events [4,8]. Glycemic control must be maintained to prevent complications following exercise for someone managing type 1 diabetes mellitus. 

Research studies that have examined the relationship between glucose variability and physical activity among adults with type 1 diabetes mellitus have reported similar findings to this study. One such study by van Dijk et al. reported a significant difference in glucose variability with a single walking event resulting in an increased variability compared to habitual physical activity [19]. The population in the current investigation was also habitually active possibly showing a similar finding. In contrast, other investigations have shown conflicting results. In a recent investigation by Brockman and colleagues, an adult sample of individuals managing type 1 diabetes mellitus showed no differences in glycemic variability and time in range on days of exercise compared to those without exercise [20]. One such reason for the conflicting findings could be the age difference and maturity levels leading to an overall better management. This could be due to the fact that adults who are habitually active and exercise regularly have a better understanding of how to maintain adequate glucose control after having had more time and life experience in doing so. It also may be due to differences in hormonal status which is a limitation of the current study due to the age range. Further, the current study did not include group comparisons on exercising days and non-exercising days which was the design of the study previously described. 

When comparing our findings to those of other research studies that included a young adolescent population, only one also explored changes in glucose values following two types of structured exercise that included continuous moderate-intensity exercise and high-intensity interval training. Results from the investigation showed that compared to continuous moderate-intensity exercise, high-intensity interval training led to a lower rate of change in glucose values with a significant difference between the two protocols [21]. A major difference between the investigations is the data collection period which ended after 30 min post-exercise, whereas the current study included glucose variability results over a period of two weeks. Whether similar results would have been reported remains unknown; however, other intervention studies in a controlled environment have shown similar results when it comes to intermittent exercise at higher intensities having less of a decline in glucose values and remaining more stable throughout the day. 

Another study completed by Singhvi and colleagues included a similar cross-sectional approach to identify associations between physical activity and glucose control which reported similar findings to that of the present study. Their investigation measured fitness levels through graded exercise testing in a sample of 19 adolescents with type 1 diabetes mellitus and found that glycemic variability was inversely associated with fitness levels in adolescents with type 1 diabetes mellitus [22]. Different indices of physical activity variables may have been used in the methods, but as in the current investigation, all participants wore a CGM and it has been well established that individuals of all ages who participate in more daily physical activity at moderate to vigorous intensity levels over the course of multiple weeks will have an increased fitness level compared to those who accumulate less.

The results from this investigation are not without limitations. It should be noted that the findings reported included mixed treatment methods for administering insulin and a small sample size of habitually active adolescent youth making it difficult to generalize the findings to those who are primarily sedentary or new to increased physical activity behaviors and/or exercise. In addition to the small sample, this study did not include a specific intervention or comparison group making it impossible to determine any cause-and-effect conclusions. There are also interfering factors not incorporated in our analysis that includes individual approaches to carbohydrate intake/insulin adjustment, and due to not having sleep data from all participants to include in the analysis we were unable to account for sleep quality which may have impacted the current findings. Further research needs to be completed to better understand the glucose response in a more controlled environment, as well as comparing individuals with a history of exercise and/or routine physical activity to those who are starting a workout regimen for the first time. Although the benefits of daily moderate to vigorous physical activity have clearly been established, whether it would be safe in certain populations managing type 1 diabetes remains less clear due to the uncertainty of the glucose response which are often determined by pre- and post-exercise variables including current glucose values, recent carbohydrate intake, and insulin adjustments.

This novel study examined more in-depth total time spent in each intensity level of physical activity independent of one another as well as combined. To our knowledge, this is the first investigation to explore the relationship of specific levels of intensity on glucose variability over a two-week period in a habitually active adolescent population with type 1 diabetes mellitus. The significant relationships observed in total duration of moderate and vigorous physical activity combined were stronger than that of moderate-intensity physical activity and/or vigorous intensity only. Results further indicated that the amount of combined moderate- and vigorous-intensity activity accumulated over a two-week period predicted lower glucose variability. It was also observed in this study that vigorous-intensity physical activity by itself did not have a significant relationship with glucose variability, whereas increased amounts of moderate-intensity physical activity showed a significant inverse relationship. This would suggest that accumulating more moderate-intensity physical activity with minor amounts of vigorous intensity combined may not only be safer, but also could be more effective way for children with type 1 diabetes mellitus to help improve daily fluctuations in circulating glucose by lowering overall glucose variability. However, it is important to note that additional research with larger sample sizes and controlling more potential confounders are necessary before any definitive statements about this relationship can be made.

In conclusion, these data demonstrated that the total amount of daily physical activity is important when properly managing type 1 diabetes mellitus, but time spent engaging in moderate and vigorous physical activity intensity levels combined could potentially have a greater influence on glucose variability during and following activity. Although more research is needed to strengthen these findings, it could be of great benefit for individuals managing diabetes to incorporate an activity monitor into their daily routine to share with their diabetes care team to better understand how physical activity levels impact their blood glucose.

## Figures and Tables

**Table 1:** Demographic characteristics.

Characteristics	N = 10
Age, years	14 ± 3
Gender	Male: 6 Female 4
Race/Ethnicity	White: 8 Black: 1 Latino: 1
T1D Diagnosis (Months)	74.22 ± 47.61
Insulin Pump Use	6 (60%)
Glucose Variability (Avg)	36.64% ± 4.66
Daily Steps	10,632.35 ± 2357.45
Vigorous Activity (mins.)	23.46 ± 18.24
Moderate Activity (mins.)	41.33 ± 15.29
Light Activity (mins.)	240.48 ± 26.54
Sedentary (mins.)	803.33 ± 126.74

Abbreviations: T1D = Type 1 Diabetes.

**Table 2:** Linear Regression Model.

Model: Simple Linear Regression Dependent Variable: Glucose Variability			
Variable	β	SE B	*p* (95% CI)
Intercept	43.23	2.72	
Moderate and Vigorous Intensity	−0.12	0.04	0.04 (−0.23–−0.01)

Note: R^2^ = 0.71; *p* < 0.01; *N* = 10. Controlled for sedentary and light intensity minutes, age, and gender.

## Data Availability

The data presented in this study are available on request from the corresponding author.
